# Recent Advanced on the MXene–Organic Hybrids: Design, Synthesis, and Their Applications

**DOI:** 10.3390/nano11010166

**Published:** 2021-01-11

**Authors:** Cheng-Feng Du, Xiangyuan Zhao, Zijiao Wang, Hong Yu, Qian Ye

**Affiliations:** 1 State Key Laboratory of Solidification Processing, Center of Advanced Lubrication and Seal Materials, Northwestern Polytechnical University, Xi’an 710072, Shaanxi, China; cfdu@nwpu.edu.cn (C.-F.D.); xiangyuanzhao@mail.nwpu.edu.cn (X.Z.); 18234119370@163.com (Z.W.); 2 Key Laboratory of Materials Processing and Mold, Zhengzhou University, Ministry of Education, Zhengzhou 450001, China

**Keywords:** MXene, 2D materials, organic hybrids, energy storage

## Abstract

With increasing research interest in the field of flexible electronics and wearable devices, intensive efforts have been paid to the development of novel inorganic-organic hybrid materials. As a newly developed two-dimensional (2D) material family, MXenes present many advantages compared with other 2D analogs, especially the variable surface terminal groups, thus the infinite possibility for the regulation of surface physicochemical properties. However, there is still less attention paid to the interfacial compatibility of the MXene-organic hybrids. To this end, this review will briefly summarize the recent progress on MXene-organic hybrids, offers a deeper understanding of the interaction and collaborative mechanism between the MXenes and organic component. After the discussion of the structure and surface characters of MXenes, strategies towards MXene-organic hybrids are introduced based on the interfacial interactions. Based on different application scenarios, the advantages of MXene-organic hybrids in constructing flexible devices are then discussed. The challenges and outlook on MXene-organic hybrids are also presented.

## 1. Introduction

Recently, with the great potential in the fields of intelligent sensing, health monitoring, and portable display, ever-growing interests have been paid into the field of flexible electronics and wearable devices [1,2,3,4]. However, the fabrication of flexible electronics is usually based on printed electronics technology, as the traditional silicon-based semiconductors have become increasingly difficult to meet the manufacturing requirements. Therefore, increasing research interests have been paid to developing relevant materials in recent years. Instead of the rigid-integrated silicon-based semiconductors, nanotechnology points out a new way towards inorganic flexible devices, which is to buffer the macroscopic deformation by the relative movement of numerous boundaries. At present, the thin-film device constructed by two-dimensional (2D) materials represents one of the most promising candidates [5]. However, the fatigue and phonon scattering at grain boundaries are still unsolved issues [6]. On the other hand, the organic semiconductors built on π-conjugated systems are well-known for their good flexibility but suffer from insufficient carrier mobility and aging resistance [7]. Therefore, the combination of advantages from both inorganic and organic materials, namely, the inorganic-organic hybrids, becomes the most obvious choice.

To obtain an ideal material that can fulfill the requirements of electrical, mechanical, and processing properties for printable electronics, the interfacial compatibility between the inorganic and organic components becomes critical [8]. For one thing, a well-compatible interface endows the two components good dispersity, which further affects the uniformity of the physicochemical properties in the final products. For another, the well-compatible interface is closely related to the carrier transportation between the inorganic and organic parts [9]. However, as the traditional 2D materials are stacked via van der Waals interaction (e.g., transition metal dichalcogenides (TMD), graphene, and phosphorene), it is not easy to introduce organic modifiers on their surface since there is rarely dangling-bond [10,11]. Since 2011, a novel 2D metal carbide/nitride family knows as MXenes, has aroused great attention in many fields owing to their high conductivity, high in-plane stiffness, and abundant surface terminal groups [12,13]. Different from other 2D analogs, the MXenes are generated from MAX phase ceramics by etching certain metal layers. In other words, the process produces a large number of surface dangling-bonds [14,15]. The surface dangling-bonds are temporarily deactivated by the T groups, whereas in early studies, the T groups can be a bridge to surfactant adsorption or directly replaced by coupling agents. However, although the organic species have been widely used to dilate the interlayer space of MXenes, adjust the surface hydrophobicity, and bringing active atoms, the detailed interaction and working mechanism of the organic species in these hybrids are rarely concerned.

In recent years, many comprehensive reviews have been made on the MXenes, MXene-based heterostructures, and hybrids. However, most of them are still mainly focused on the applications [16,17,18,19,20,21], less attention paid to the surface adjustability and the interfacial compatibility of the MXene-organic hybrids. In this consideration, we try to briefly summarize the recent progress on this material system, thus offer the readers a deeper understanding of the interaction and collaborative mechanism between the MXenes and organic component. In the first part, the structure and surface characters of MXenes are briefly introduced. In the second part, strategies towards MXene-organic hybrids are discussed based on interfacial interactions. The advantages of MXene-organic hybrids in constructing flexible devices are discussed in the third part, which is classified by different application scenarios. Finally, the challenges and outlook on MXene-organic hybrids are presented.

## 2. Structure and Surface Characters of MXenes

**Figure 1:**
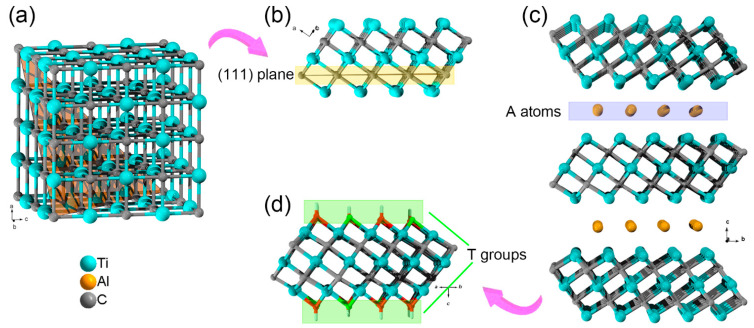
(**a**) A typical *fcc* lattice of TiC. (**b**) The extracted layer from *fcc* TiC lattice along (111) plane. (**c**) The side view of Ti_3_AlC_2_ lattice and (**d**) a monolayer of Ti_3_C_2_T*_x_* MXene.

The MXenes are a kind of 2D metal carbide/nitride with an alternative atomic arrangement of M_*n*+1_X*_n_* (M = transition metals; X = C, N; *n* = 1, 2, 3, 4). Take the Ti_3_C_2_T*_x_* MXene as an example. The Ti_3_C_2_ skeleton can be regarded as an extracted layer from *fcc* metal carbides lattice along (111) plane (Figure 1). Since the first discovery of Ti_3_C_2_T*_x_* MXene in 2011, more than 70 MXenes have been theoretically predicted or synthesized. As mentioned above, the synthesis of MXene follows a “top-down” protocol: selective etching of the A atoms from MAX phase ceramics. However, as the M*_n+1_*X*_n_* layers and A atoms are alternatively arranged in MAX phase ceramics, the removal of A atoms will break M-A bonds, thus results in high-active M atoms at the surface. As first presented by Gogotsi et al., the surface terminal groups (T groups, e.g., –F, –O, or –OH) are necessary for stabilizing MXenes due to the high activity of these coordination unsaturated surface metal atoms [22]. Furthermore, owning to these surface terminal groups, the MXenes with typical inorganic carbide skeleton usually presents similar surface physicochemical properties (e.g., hydrophilicity and oxidizability) [23].

Attributing to the surface T groups, the fresh MXene flakes are negatively charged and highly hydrophilic, which will form the colloidal dispersion by surface electrostatic repulsion. Based on the double electrical layer theory, it is easy to speculate that the dispersity of MXene flakes will change along with different solution conditions. Also depends on the average oxidation states of the M and X elements, the Zeta potential of MXenes is varied. In the past several years, the oxidation of MXenes in water also arouses wide concerns, which is found to be isogenesis with their hydrophilic. Still exampled by Ti_3_C_2_T*_x_* MXene, although TiO_2_ species are detected when annealing the MXene under either vacuum, inert atmosphere (Ar, N_2_), or oxidizing atmosphere (CO_2_, air), the oxidation can be dramatically slowed down by decrease the environment temperature. However, in an aqueous suspension, nearly half of the flakes degrade in five days, even at room temperature [24,25]. The degradation behavior of MXenes in the aqueous suspension then can be ascribed to the water or the synergy of water and dissolved oxygen molecules. Interestingly, Kathleen et al. found that with oxygen solubility orders of magnitude higher than water, the organic solvent (N,N-dimethylformamide (DMF), 1-methyl-2-pyrrolidinone (NMP), propylene carbonate (PC), and ethanol) did not cause observable oxidation of MXenes [26]. Therefore, a deeper insight into the relationship between the surface T groups on Ti_3_C_2_T*_x_* MXene and their dispersion properties was presented, namely, the surface tension, polarity index, dielectric constant (*ε*), and cohesive energy density (*δ*). However, although some of the organic solvents were screened out to be a good medium for Ti_3_C_2_T*_x_* MXene dispersion, the dispersibility of Ti_3_C_2_T*_x_* MXene in nonpolar organic solvents is still an unsolved issue. What is more, the T groups usually have a random constituent and distribution, which makes the determination of surface properties more difficult.

Enlightened by traditional strategies towards surface compatibility regulation, the surface physicochemical properties of MXenes also can be dramatically altered by replacing the inorganic surface terminal groups with organic species. For example, di(hydrogenated tallow)benzyl methyl ammonium chloride (DHT)-treated Ti_3_C_2_T*_x_* MXene has shown a good dispersibility in nonpolar organic solvents [27]. More importantly, the study opens up a new way for MXene-polymer hybrids, which have good interfacial compatibility. In the following section, based on the interaction between organic species and MXenes, the synthesis and corresponding surface physicochemical properties of MXene-organic hybrids will be briefly discussed.

## 3. Strategies towards MXene-Organic Hybrids

### 3.1. MXene-Organic Hybrids through Covalent Interaction

Benefiting from the surface atomic layers of early transition metals with empty *d* orbital, the MXenes skeleton usually presents a strong affinity to electron donors. When the organic molecules with electron-donating groups are introduced, the original T groups can be replaced. To date, numerous small organic molecules are found to be able to covalently connect on MXenes.

**Figure 2:**
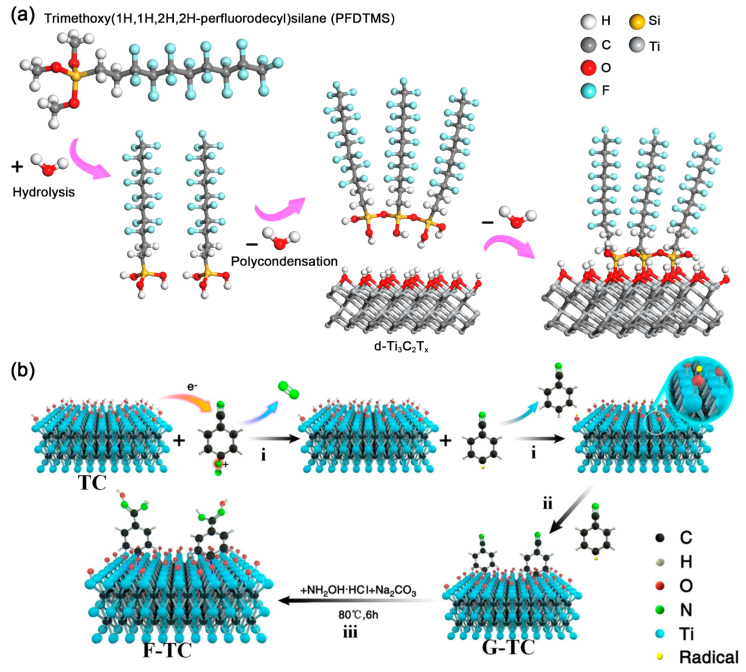
(**a**) Surface modification process of d-Ti_3_C_2_T*_x_* nanosheets by PFDTMS; Reproduced with permission [29]. Copyright 2018, Royal Society of Chemistry. (**b**) Proposed mechanism of amidoxime functionalization for MXene Ti_3_C_2_T*_x_*; Reproduced with permission [38]. Copyright 2020, American Chemical Society.

Silane (RSiX*_n_*), one of the most commonly used organic coupling agents, has been widely applied for surface modification of inorganic materials. For silane, the R represents the functional group, such as an amino, alkane, aromatic group, epoxy group, and so on; X stands for hydrolysis group, containing Cl, OMe, OEt, etc. [28]. During the so-called “hydrolytic condensation” process (Figure 2a), X groups will first be hydrolyzed to form a siloxane (–SiOH) structure. Later, the siloxane goes through a dehydration condensation reaction to form oligomers. The –OH groups in oligomers will react with –OH groups on MXenes, forming an intermediate state. Finally, the Ti–O–Si covalent bonds are formed by dehydration condensation [28,29]. Meanwhile, different properties inherited from *R* groups can be introduced into the inorganic phase. In 2018, Zhao et al. reported a trimethoxy (1H,1H,2H,2H-perfluorodecyl) silane (PFDTMS) modified Ti_3_C_2_T*_x_* MXene membrane prepared via a simple wet route [29], in which the water contact angle increased from 38.8° to 102.0° after PFDTMS grafting. Later, Sehyeong et al. have systematically studied the surface polarity regulation of Ti_3_C_2_T*_x_* MXene by silane with different R groups. After replacing the surface T groups of Ti_3_C_2_T*_x_* MXene with lipophilic octyltriethoxysilanes (OTS) [30], the OTS@MXene showed a water contact angle of 102.6° and exhibited excellent dispersion stability in non-polar hexane for more than four weeks. When methyltriethoxysilane (MTS), propyltriethoxyislane (PTS), and hexyltriethoxysilane (HTS) was selected, the surface hydrophobicity of these silane-MXene hybrids would decrease with shortening the alkyl chain. Beyond the randomly surface grafting, Zhang et al. designed a well-ordered vertically aligned Janus MXene-based aerogel, which has different wettability belong to two sides named VA-MXA [31]. After modifying the Ti_3_C_2_T*_x_* MXene by fluorinated alkyl silane under vacuum conditions, the water contact angle of the upper layer increased from 44.6° to 133.2°. Also, the excessive concentration of silane clogged the available surface and decreased the hydroxyls on the surface of Ti_3_C_2_T*_x_*, which can bring novel surface physicochemical properties [28]. In Tran and his co-workers’ work [32], the N,N-(diethylamino)dithiocarbamoyl-benzyl(trimethoxy) silane (SBDC) was used to functional Ti_2_CT*_x_*. Interestingly, the SBDC-MXene hybrid was applied as both substrate and iniferter, which allowed further polymerization of thermoresponsive polymer (poly(2-(dimethylamino)ethyl methacrylate (PDMAEMA)) on the surface of Ti_2_CT*_x_*_._ On the other hand, the silane molecules can not only change the surface hydrophilcity, but also alter the surface charged state (zeta potential) of MXenes. For instance, the aminosilane with positively charged amino group was demonstrated to have a critical role on the surface charge of aminosilane-Ti_3_C_2_T*_x_* hybrids [33,34]. As reported by Hossein et al. the [3-(2-aminoethylamino)propyl]-trimethoxy silane (AEAPTMS)-Ti_3_C_2_T*_x_* hybrids had a positive zeta potential of +62 mV at pH = 2.58, which is the highest zeta potential reported for MXenes to date. Similar to silane, the alkylphosphoric acid can also form covalent bonds with MXene through dehydration condensation reaction. Kim et al. demonstrated a simultaneous interfacial chemical grafting and phase transfer method for alkylphosphoric acid (C*_n_*PA, *n* = 3, 6, 8, 10, 12) grafting on the Ti_3_C_2_T*_x_* flakes [35]. During the reaction, Ti–O–P bonds formed by interfacial nucleophilic addition and sequential condensation reaction between hydroxyl groups from Ti_3_C_2_T*_x_* and phosphate group from C*_n_*PA. The pH and concentration of C*_n_*PA are found to influence the interfacial chemical reaction and phase transfer. Whereas the dispersion stability of the Ti_3_C_2_T*_x_*-C*_n_*PA hybrids in nonpolar organic medium can be attributed to the steric stabilization and strong nonpolar interaction of long alkyl chains. Very recently, Sun et al. proposed new evidence on the existence of hydrogen bonds between Ti_3_C_2_T*_x_* nanosheets and C_8_PA [36]. Therefore, it seems that the surface interaction between MXenes and coupling agents may be more complicated than we think.

Diazonium salt is another commonly used reagent to covalently connect organic functional groups on MXenes. In 2020, Zhang et al. first proposed a detailed reaction mechanism between aryl diazonium salts and Ti_3_C_2_T*_x_* MXene, in which the aromatic primary amine can undergo a diazotization reaction and the produced diazonium salts will covalently bond with MXenes. As shown in Figure 2b, the detailed reaction mechanism can be divided into three steps [37,38]: (1) an electron was transferred from Ti_3_C_2_T*_x_* MXene to aryl diazonium salt, which resulted in the cleavage of diazonium to nitrogen and aromatic free radical; (2) the aromatic free radical received an additional H atom from Ti_3_C_2_T*_x_* MXene, generating a Ti–O radical on the surface of MXene; (3) strong Ti–O–C covalent bonds formed between Ti–O radical and aromatic free radical. In fact, sulfanilic acid diazonium salts were introduced to get large-scale delaminated Ti_3_C_2_T*_x_* multilayers as early as 2016 [39,40,41]. Compared with the pristine Ti_3_C_2_T*_x_*, the phenylsulfonic acid grafted Ti_3_C_2_T*_x_* MXene becomes amphiphilic material, which shows an increased solubility and excellent stability in water for more than one month. However, in consideration of the potential aromatic π interaction and the oxygen from the sulfonic group, the works did not give a detailed mechanism and solid evidence on the formation of MXene-aryl (Ti–O–C) linkages. Recently, Muhammad et al. compared the noncovalent and covalent interaction mechanism of 1-aminoanthraquinone (AQ) on Ti_3_C_2_T*_x_* MXene (denoted as AQ@Ti_3_C_2_T*_x_* and AQ-Ti_3_C_2_T*_x_*, respectively) [42]. It is found that after the diazonium reaction, the amino group disappeared, and the covalent bond formed between AQ and Ti_3_C_2_T*_x_*. As observed, the covalently attached AQ on Ti_3_C_2_T*_x_* MXene offered better charge transportation, thus electrochemical performance. According to the abovementioned works, the diazonium reaction can be a useful way for the covalent connection of organic molecules onto the MXene nanosheets and further expands the applications of MXene-organic hybrids.

Taking advantage of the electrical conductivity and layered structure of MXenes, the MXene-polymer hybrids usually show improved properties when compared with the pure polymer, such as mechanical properties [43,44,45,46], flame retardancy [47,48], and responsiveness [32,49]. In early 2015, an MXene-organic hybrid with CO_2_ and temperature dual-responsive property was reported by Chen et al. [49]. The hybrid consisted of V_2_CT*_x_* MXene and self-initiated photo grafting and photopolymerized (SIPGP) poly(2-(dimethylamino)ethyl methacrylate) (PDMAEMA) brushes. The hydroxyl groups on V_2_CT*_x_* MXene were acting as the photoactive sites, which allowed further growth of polymer brushes via SIPGP. When pre-modified MXene by SBDC [32], PDMAEMA brushes can also be connected onto the MXene surface. Zhang et al. [50] utilized in situ free radical polymerization to prepare Ti_3_C_2_T*_x_*/polyacrylamide (PAA) hybrid with acrylamide monomer and potassium persulfate as redox initiator, in which the Ti_3_C_2_T*_x_* MXene was introduced as the crosslinker.

### 3.2. MXene-Organic Hybrids through Electrostatic Interaction

In addition to utilizing T groups of MXenes to covalently graft organic molecules by chemical reactions, the electrostatic interactions on negatively charged MXenes surface can also be utilized. Cui et al. synthesized the (3-aminopropyl) triethoxylsilane (APTES) grafted Si nanoparticles first [51], in which the covalently X groups on silane were consumed. However, by directly using the positively charged Y group (here, amino), the grafted Si nanoparticles were still self-assembled with Ti_3_C_2_T*_x_* through electrostatic interaction. Xu et al. reported microcontact printing to get ultrathin MXene micropatterns [52]. The MXene ink was applied to a polydimethylsiloxane (PDMS) film with patterns of alternating grooves. Subsequently, transfer printing it onto APTES-modified coverslips. In contrast, Ti_3_C_2_T*_x_* adsorbed onto coverslips by electrostatic interaction with amino groups.

**Figure 3:**
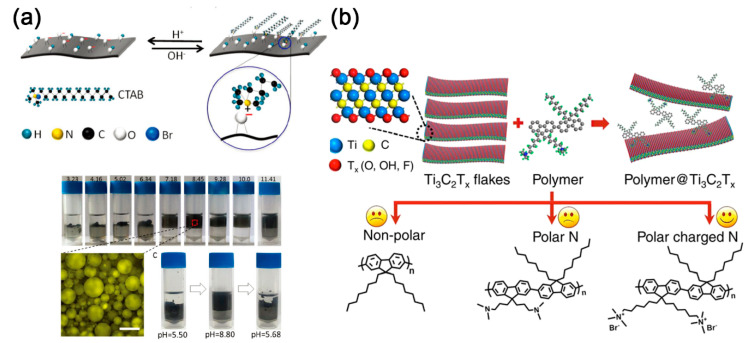
(**a**) Top: a possible scheme describing the interaction between cetyltrimethylammonium bromide (CTAB) and Ti_3_C_2_T*_x_* MXene. Bottom: the emulsion stability as a function of the pH of the aqueous phase at a fixed weight ratio of CTAB to MXene; reproduced with permission [56]. Copyright 2018, Royal Society of Chemistry. (**b**) Schematic illustration of the interaction of the polymer with Ti_3_C_2_T*_x_* layers (top) and the synthesized polymers with nonpolar, polar, and polar charged nitrogen lateral chain ends (bottom); Reproduced with permission [57]. Copyright 2017, American Chemical Society.

As another kind of common-used organic molecules, alkylammonium salts including dodecyl trimethyl ammonium bromide (DTAB), tetradecyltrimethylammonium bromide (TTAB), cetyltrimethylammonium bromide (CTAB), stearyl trimethyl ammonium bromide (STAB), octadecyl trimethyl ammonium bromide (OTAB), dioctadecyl dimethyl ammonium chloride (DDAC), dihexadecyl dimethyl ammonium bromide (DDAB), and DHT are usually used to insert in MXene layers through electrostatic adsorption. After pillared by alkylammonium, the Ti_3_C_2_T*_x_* MXene presented a dramatic increase in interlayer spacing [53,54]. Obviously, the interlayer spacing increased along with the increasing length of alkyl chains [55]. Bian et al. proposed that the positively charged ammonium group might mainly be anchored on the surface –O groups from Ti_3_C_2_T*_x_* MXene [56], and the intercalation of alkylammonium can also affect the wettability of MXene (Figure 3a). Maybe because of the competitive adsorption of cations, the CTA cation pillared Ti_3_C_2_T*_x_* MXene can only form a stable oil-in-water emulsion in neutral or basic condition. While in acidic conditions, the MXene will aggregate at the oil/water interface. In a current study reported by Michael et al., the DHT not only improved the organophilic nature of Ti_3_C_2_T*_x_*, produced stable colloidal suspensions of Ti_3_C_2_T*_x_* in nonpolar solvents but also shielded the MXene flakes from oxidation in water [27].

Amino acids are also studied as the organic part towards MXene-organic hybrids. Elumalai et al. proposed that the electrostatic interaction between –OH and –F groups on Ti_3_C_2_T*_x_* MXene and the zwitterion consisted of amino acid molecules might be the driving force for the spontaneous intercalation [58]. The MXene hybrids contained negatively charged glycine (Gly), phenylalanine (Phe), tryptophan (Trp), and histidine (His) can form stable suspensions in water due to the interparticle electrostatic repulsion. Whereas aromatic (Phe, Trp, His) intercalation may also produce rutile TiO_2_@Ti_3_C_2_T*_x_* by sonication. On the contrary, combined with first principle calculations (DFT), recently, Chen et al. studied the interaction between Gly and double-layer structure Ti_3_C_2_O_2_ [59]. From their analysis, the Ti and N atoms from Ti_3_C_2_O_2_ and Gly, respectively, were likely to share electrons and lead to the formation of Ti–N bonding. Obviously, the ionizable organic molecules are another good choice for preparing MXene-organic hybrids with controllable properties. However, the unclear interaction mechanism makes it an urgent task to be solved.

Carey and his coworkers prepared the 12-aminolauric acid (ALA), or DHT-modified Ti_3_C_2_T*_x_* first (denoted as ALA-Ti_3_C_2_T*_x_* and DHT-Ti_3_C_2_T*_x_*) [60,61]. After the modification, MXene changed from hydrophilic to organophilic, so the intercalation of the ε-caprolactam monomer and 6-aminocaproic acid catalyst was easier [60]. Additionally, uniform Ti_3_C_2_T*_x_* MXene/epoxy hybrids can be synthesized by ALA-Ti_3_C_2_T*_x_* and DHT-Ti_3_C_2_T*_x_*. The authors proposed that ALA and DHT can speed up the curing reaction of epoxy in the interlayer space, which further enlarged the interlayer space of the MXene layers [61].

**Figure 4:**
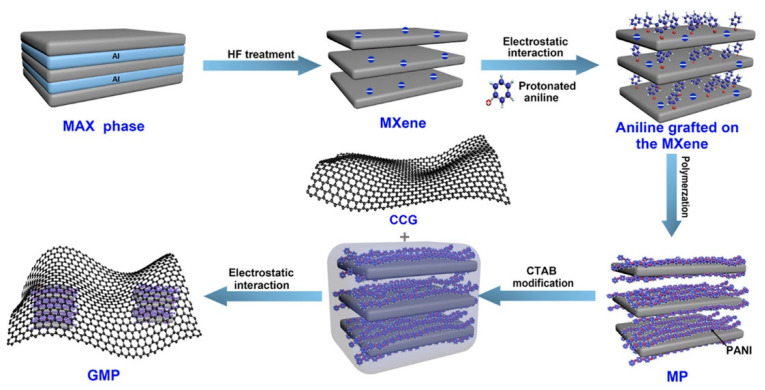
Scheme of the synthesis of graphene-encapsulated Ti_2_CT*_x_*@PANI hybrid (GMP); Reproduced with permission [63]. Copyright 2018, American Chemical Society.

Similarly, the aniline monomers are believed to electrostatically adsorb on and between the multilayer MXene nanosheets [62]. Fu et al. reported a graphene-encapsulated Ti_2_CT*_x_*@polyaniline (PANI) hybrid named GMP for supercapacitors [63]. The adsorbed aniline monomers can be chemical oxidative polymerized with ammonium persulfate as the oxidant to form Ti_2_CT*_x_*@PANI hybrid (Figure 4). For graphene encapsulation, CTAB was utilized to lower the surface energy and change the zeta potential of Ti_2_CT*_x_*@PANI. Recently, there is also evidence that the surface functional groups on MXenes will facilitate nucleation during the PANI polymerization [64].

When turning to the other common-used conducting polymer, poly(3,4-ethylene dioxythiophene) (PEDOT), Gogotsi and his co-workers revealed the mechanism of charge-transfer-induced polymerization of EDOT on the surface of Ti_3_C_2_T*_x_* MXene [65]. Verified by the theoretical calculations, the parallel orientation of EDOT monomer on Ti_3_C_2_T*_x_* surface presented the most stable configuration with the lowest binding energy of −1.02 eV. After adsorption, 0.34 electrons were transferred from EDOT to Ti_3_C_2_O_2_, which initiated the in situ polymerization of EDOT on Ti_3_C_2_O_2_. With the energy supplement via electrochemical reaction, Qin et al. demonstrated a one-step in situ electrochemical polymerization for Mo_1.33_C/PEDOT and Ti_3_C_2_T*_x_*/PEDOT films with controlled thickness and micropattern [66]. However, due to the insoluble nature of PEDOT, the commercial product usually contains a mixture of poly(styrene sulfonate) (PSS). In consideration of the bicomponent structure and the ionic chain nature, the ex-suit blending method was more popular than in-suit polymerization towards MXene-PEDOT:PSS hybrids and the electrostatic interaction between PSS and MXene promoted the formation of 3D interconnected and reticulated structure. The hydrogen from –SO_3_H on PEDOT:PSS with –OH,–F on Ti_3_C_2_T*_x_* and the electrostatic interaction between PSS and MXene were also found to jointly shield the coulombic attraction between PEDOT and PSS, which enhanced the inter-chain charge transport between PEDOT chains [67]. The Ti_3_C_2_T*_x_*/(PEDOT:PSS) film can be fabricated by drop-casting [68], vacuum-assisted filtration [69], and simply filtration [70,71]. Recently, MXene/(PEDOT:PSS) hybrid was also fabricated into a fiber-shaped supercapacitor by a one-step wet-spinning approach [72].

By the ex-situ blending method, plenty of MXene/polymers hydrides can be prepared. Firstly reported by Gogotsi et al., charged poly-diallyl dimethyl ammonium chloride (PDDA) and electrically neutral polyvinyl alcohol (PVA) were blended with Ti_3_C_2_T*_x_* by vacuum-assisted filtration [73]. Subsequently, the dispersibility of MXene sheets in the PVA matrix and the dielectric performance of MXene/PVA hybrids in X-band frequency was studied [74]. Boota et al. synthesized polyfluorene (PFO) derivatives containing nonpolar, polar nitrogen-containing, and charged nitrogen-containing lateral chains by Suzuki polycondensation reaction (Figure 3b) [57]. They have pointed out that both the electrostatic interactions and hydrogen bonds may be involved between MXene and PFO derivatives. However, the bulky methyl groups from polar polymers with charged nitrogen-containing ends prohibited the formation of the hydrogen bond. A physical vapor deposition technique, resonant infrared matrix-assisted pulsed laser evaporation (RIR-MAPLE), was performed by Ajnsztajn et al. to produce Ti_3_C_2_T*_x_*/PFO transparent hybrid electrode [75]. Via RIR-MAPLE, the film exhibited the minimal phase segregation.

### 3.3. MXene-Organic Hybrids through Hydrogen Bonds and Other Supermolecular Interactions

**Figure 5:**
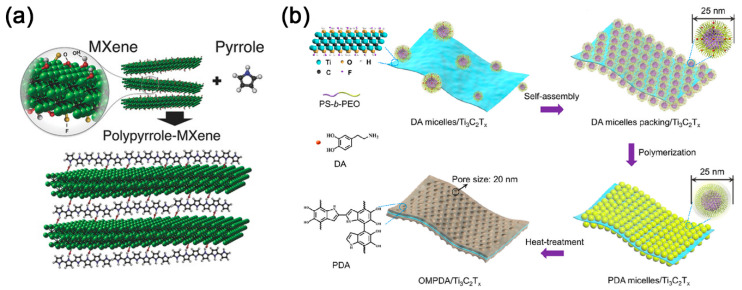
(**a**) Schematic illustration of pyrrole polymerization using MXene; reproduced with permission [76]. Copyright 2016, WILEY-VCH. (**b**) Schematic drawing depicting the preparation steps of OMPDA/Ti_3_C_2_T*_x_* hybrid: (i) mixing the DAmi with Ti_3_C_2_T*_x_* nanosheets, (ii) packing of DAmi and subsequent, (iii) direct polymerization of polydopamine (PDA) micelles on the surface of Ti_3_C_2_T*_x_* nanosheets, and (iv) obtaining the OMPDA/Ti_3_C_2_T*_x_* after heat treatment; Reproduced with permission [81]. Copyright 2020, American Chemical Society.

Except for the strong inorganic-organic correlation provided by covalent and electrostatic interaction, the supermolecular interactions are also critical to the formation of MXene-organic hybrids. In 2016, a Ti_3_C_2_T*_x_*/polypyrrole (PPy) hybrid with highly aligned PPy chains in between the Ti_3_C_2_T*_x_* layers was prepared by unconventional oxidant-free polymerization (Figure 5a) [76]. Specifically, the key factor in the alignment process was the hydrogen bond, which might originate from the N–H group of the pyrrole ring and terminating oxygen or fluorine on the Ti_3_C_2_T*_x_* surface. What is more, the fluorine on Ti_3_C_2_T*_x_* will be doped in PPy chains, which can further enhance their electrochemical activity. Different from the oxidant-free chemical polymerization, Zhu et al. [77] fabricated Ti_3_C_2_T*_x_*/PPy hybrid film via electrophoretic deposition and electrochemical polymerization. The pronounced acidic character of the Ti_3_C_2_T*_x_* surface generated hydrogen bonding between Ti_3_C_2_T*_x_* and PPy during electrochemical polymerization. However, for the most traditional pyrrole polymerization [78], FeCl_3_·6H_2_O was applied as an oxidant, and the strong oxidizing Fe^3+^ ions will result in the oxidation of Ti_3_C_2_T*_x_*. Therefore, Zhang et al. only found the 3D TiO_2_@NC/Fe_7_S_8_ hybrids by in situ polymerization of pyrrole monomer with alkalized Ti_3_C_2_T*_x_*.

As confirmed by previous studies, quinones are important redox-active centers for the chemical reaction [79]. However, there are only a limited amount of quinones reserved in polydopamine (PDA), which is positively correlated to the oxidation state of PDA [80]. Interestingly, Ti_3_C_2_T*_x_*/PDA hybrids were found to be an excellent precursor for o-benzoquinone after low-temperature heat treatment at 300 °C [79]. After that, Li et al. further prepared vertically oriented ordered mesopores (OM) PDA/Ti_3_C_2_T*_x_* hybrid (OMPDA/Ti_3_C_2_T*_x_*) by using the PS-b-PEO block polymer as a soft-template (Figure 5b) [81]. In their report, the hydrogen bond also played a crucial role in the assembly of dopamine/PS-b-PEO micelles and Ti_3_C_2_T*_x_*. Recently, Du and coworkers utilized Ti_3_C_2_T*_x_*@PDA prepared by in situ polymerization as starting materials to hybrid with poly(ethylene glycol) (PEG)-based polyurethane (PU) [82]. The PDA is found to be a medium molecule that facilitated the interfacial compatibility of Ti_3_C_2_T*_x_* flakes in PEG-based PU, where the Ti_3_C_2_T*_x_*@PDA covalently bonded with PU during the process of copolymerization.

Beyond anchoring the organic species on the MXene surface, the hydrogen bond also functions as a flexible junction between the inorganic MXene and organic part. Hybrid consisting of Ti_3_C_2_T*_x_* and polyacrylamide (PAM) was fabricated by Niu et al. [50]. In 2020, the exfoliated Ti_3_C_2_T*_x_* nanosheets were reported for the first time that acted as a crosslinker instead of traditional organic molecules. In the hybrid, hydrogen bonding between the –CONH_2_ groups from PAM chains and the hydrophilic groups (–OH and –F) from the Ti_3_C_2_T*_x_* nanosheets were observed. Zhang et al. simply prepared MXene hydrogel hybrids by mixing Ti_3_C_2_T*_x_* MXene and commercial polyvinyl alcohol (PVA) hydrogel [83]. They indicated the secondary crosslinking between MXene and PVA and a dense network yielded by polymer chain entanglements. Xuan et al. improved this method by added MXene flakes into a homogeneous PVA solution [84], with the borate solution acting as the crosslinking agent, MXene bonding with PVA chains, and tetra-functional borate ion covalently. A similar interaction was also found in hydrophobically associated dry polyacrylamide (HAPAM) hydrogel [85]. In HAPAM hydrogel, hydrogen bonds between MXene and amide group of HAPAM became additional crosslinking points, which contributed to the hybrid double-network with N,N’-methylene diacryl amide (MBAA) crosslinker and affected the self-healing ability and temperature sensibility. When there were two or more different kinds of monomers or polymers together with MXene nanosheets, MXene-copolymeric hydrogels can also be prepared [86,87,88].

On the aspect of ex situ blending, Le et al. intertwined Ti_3_C_2_T*_x_* MXene particles in tangled PPy nanowires through hydrogen bond [89]. The intercalated PPy chains between the conductive Ti_3_C_2_T*_x_* sheets prevented the dense stacking of Ti_3_C_2_T*_x_*. Meanwhile, the intercalation further improved the structural stability of PPy backbones. Moreover, Ti_3_C_2_T*_x_* MXene and PANI hybrid was also prepared by mechanical blending [90]. The lightweight Ti_3_C_2_T*_x_*/PANI hybrids for EMI shielding was obtained by simply mixing PANI powder with MXene powder. The authors suggested that an equal concentration of both materials would be beneficial for enhanced interfacial interaction between them.

Except for hydrogen, the polymer chain entanglements, ionic interactions, covalent bonding were also reported, and the role of MXene sheets in hydrogel varies [86]. MXene polymer hydrogel was gradually developed with improved performance. The typical interactions between MXene and organic modified species are summarized in Table 1.

## 4. MXene-Organic Hybrids for Flexible Devices

### 4.1. MXene-Organic Hybrids for Flexible Supercapacitors

MXenes have been the center of general scientific attention since it was first synthesized in 2011 [91,92] because of their large specific surface areas, good hydrophilicity, and excellent metallic properties. It is believed that the interlayer space of MXenes can accommodate various cations, which is particularly well-suited for energy storage devices, e.g., supercapacitors and metal-ion batteries [93]. In a previous study, a high capacity of about 900 F cm^−3^ can be achieved in Ti_3_C_2_T*_x_* MXene [22], which was higher than all the reported carbon-based capacitors [31]. However, the inevitable restacking of MXene sheets inhibited the diffusion and mass transfer process and hampered the access of electrolyte to the surface area. For this purpose, enlarging the interlayer space and simultaneously keeping the contact of MXene sheets become rather important. Here, modifying the surface of the MXenes by organic species is a pursuable method, which could increase the ion adsorption and reaction sites, and further enhance their capacitive performance.

**Figure 6:**
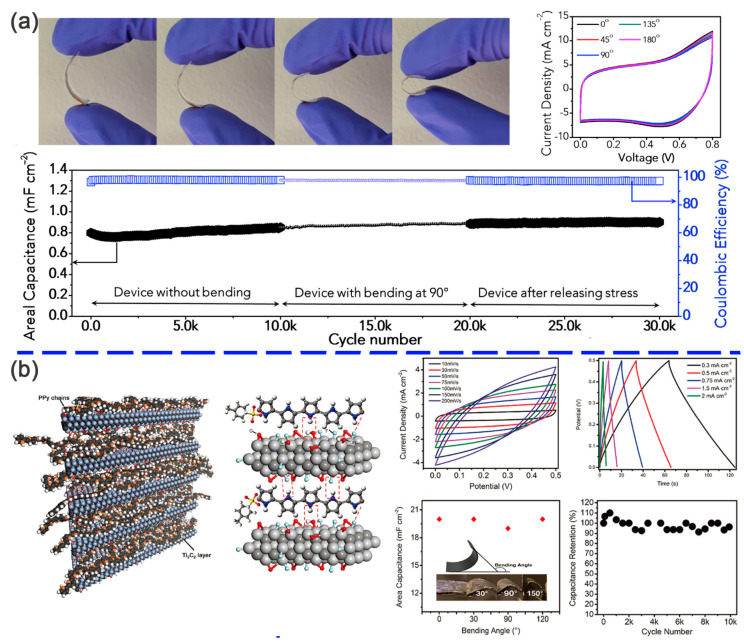
(**a**) Top: digital photographs and cyclic voltammetry curves of Ti_3_C_2_T*_x_*/PEDOT:PSS hybrids at bending angles of 0°, 45°, 90°, 135°, and 180°. Bottom: capacitance retention and coulombic efficiency of Ti_3_C_2_T*_x_*/ polymer, poly(3,4-ethylene dioxythiophene) (PEDOT): poly(styrene sulfonate) (PSS) hybrids over 30,000 cycles in the planar, bent, and released states. Reproduced with permission [94]. Copyright 2019 Elsevier. (**b**) Left: schematic of intercalated Ti_3_C_2_T*_x_*/polypyrrole (PPy) in the interlayers of l-Ti_3_C_2_T*_x_* and the hydrogen bonds between PPy and l-Ti_3_C_2_T*_x_*. Right: performance of the as-prepared solid-state supercapacitor based on the PPy/l-Ti_3_C_2_ film. Reproduced with permission [77]. Copyright 2016 WILEY-VCH.

Qin et al. evaluated the capacitive properties of the Mo_1.33_C MXene/PEDOT:PSS film [71], which presented a volumetric capacitance of 1310 F cm^−3^ at 2 mV s^−1^ in a three-electrode setup. When been fabricated into a flexible solid-state supercapacitor, the volumetric capacitance can still reach 568 F cm^−3^ at a current density of 0.5 A cm^−3^. In addition, an ultrahigh energy density of 33.2 mW h cm^−3^ and a power density of 19.47 W cm^−3^ were observed in the device. Further validated by Li et al., the insertion of conductive PEDOT chains resulted in the increasing interlayer spacing of Mo_1.33_C MXene sheets and more surface redox processes, and thus enhanced the electrochemical capacitance [70]. The conductive PEDOT chains were believed to be a bridge in forming the multidimensional electronic transport channels, which promoted the electrochemical reaction process. Gund et al. [94] prepared, flexible Ti_3_C_2_T*_x_*/PEDOT:PSS hybrids (Figure 6a) with optimal mass ratios of 1:2. Benefiting from the porous architecture, the hybrid experienced a rapid charge transport within the electroactive quinoid structure of PEDOT:PSS, and a strong surface interaction, exhibiting an areal capacitance of 2 mF cm^−2^ and volumetric capacitance of 83 F cm^−3^ at a scan rate of 1000 V s^−1^. After been assembled into a symmetric electrochemical capacitor, the areal and volumetric capacitance reached 2 mF cm^−2^ and 83 F cm^−3^ at the same scan rate with long-term durability over 30,000 cycles.

**Figure 7:**
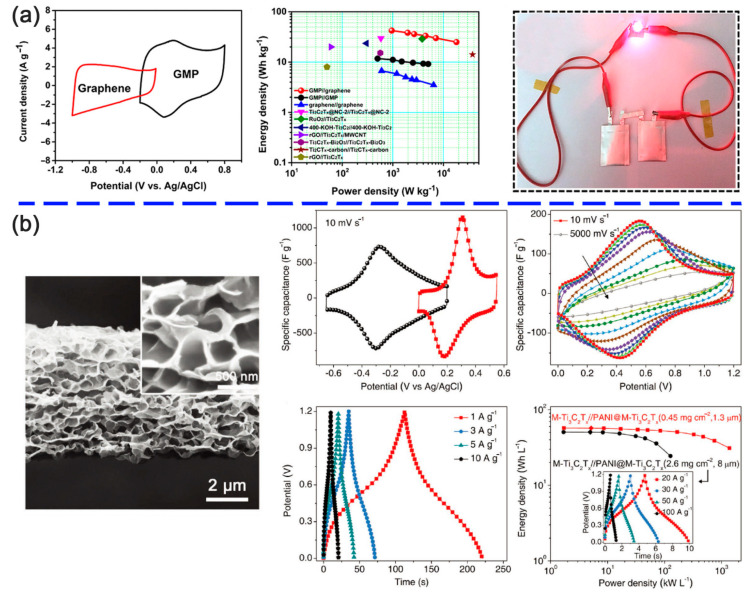
(**a**) Left: cyclic voltammetry curves of the GMP and graphene electrodes at a scan rate of 5 mV s^−1^. Middle: Ragone plot displaying the energy and power densities of the p-ASC in comparison to the symmetric device and other competitive MXene-based supercapacitors. Right: digital photo of a red LED lightening by two series-connected p-ASCs [63]. Copyright 2018 American Chemical Society. (**b**) Left: cross-sectional SEM images of 3D PANI@M-Ti_3_C_2_T*_x_* film. Right: electrochemical performance of the M-Ti_3_C_2_T*_x_*ǁPANI@M-Ti_3_C_2_T*_x_* asymmetric device. Reproduced with permission [99]. Copyright 2020 WILEY-VCH.

Besides PEDOT:PSS, the conductive polymer, such as PPy and PANI, are also widely utilized in supercapacitors [66]. Zhu et al. found that the PPy/l-Ti_3_C_2_T*_x_* film delivered an areal capacitance of 203 mF cm^−2^ when compared with that of the pristine Ti_3_C_2_T*_x_* MXene (150 mF cm^−2^) and maintained nearly 100% of the initial values after 20,000 charging/discharging cycles (Figure 6b) [77]. A symmetric solid-state flexible supercapacitor was fabricated using the PPy/l-Ti_3_C_2_T*_x_* films and PVA–H_2_SO_4_ solid-state electrolyte. As shown in Figure 6b, the capacitance was stable after 10,000 cycles, and no sharp fluctuation was found during bending. The enhanced performance was ascribed to the synergy of l-Ti_3_C_2_T*_x_* with unfolded PPy molecules and the strong hydrogen bonds between the two materials. Therefore, the hybrid of Ti_3_C_2_T*_x_* MXene and PPy chains not only improved the structural stability of PPy backbones but also provided more pathways for charge carriers. Boota et al. [76] further improved the volumetric capacitance up to 1000 F cm^−3^ by adjusting the ratio of PPy and Ti_3_C_2_T*_x_* in the hybrid film. It was found that the increase in capacitance was ascribed to the aligned PPy chains and the intercalated pyrrole molecules between the Ti_3_C_2_T*_x_* monolayers, which improved the electronic conductivity, reversibility in a redox reaction, and ion transportation. In addition, different morphology and structures of the PPy/MXene were also designed by other teams, such as a 3D Ti_3_C_2_T*_x_*@PPY nanowire network [89], organ-like Ti_3_C_2_T*_x_*/PPy [53], carambola-like MXene/PPy [95] and MXene/(BC)@PPy [96]. Xu [64] reported a Ti_3_C_2_T*_x_*/PANI hybrid as an electrode with the capacitance 556.2 F g^−1^ at 0.5 A g^−1^, which had a capacitance retention of 78.7% at 5 A g^−1^. This improvement was derived from the numerous channels for the electrolyte ions to ingress and a decreased charge transfer resistance. VahidMohammadi et al. prepared a freestanding flexible MXene/PANI electrode with a thickness of 4 μm [97]. With a mass loading of 1.34 mg cm^−2^, and the hybrid exhibited a specific capacitance of about 503 F g^−1^ at a scan rate of 2 mV s^−1^ with 98.3% capacitance retention after 10,000 cycles, validating good cycling stability. Fu et al. synthesized a graphene-encapsulated MXene Ti_3_C_2_T*_x_*@PANI (GMP) hybrid material and assembled it into an asymmetric pouch-type supercapacitor [63]. It was found that the GMP electrode exhibited excellent performance with a gravimetric capacitance of 635 F g^−1^. The cycling stability of the GMP electrode and integrated supercapacitor was 97.54% and 94.25% at 10 A g^−1^ after 10,000 cycles (Figure 7a), respectively. The robust hierarchical nanostructure and complementary synergistic effect facilitated the electrochemical process. As shown, an LED was successfully lightened by a two-series connected pouch-type GMPǁgraphene asymmetric supercapacitor (p-ASCs). Yun [98] first reported the layer-by-layer (LbL) assembly of the PANI nanofibers (PNFs)/Ti_3_C_2_T*_x_* with a composition of 77 wt % PNFs and 23 wt % MXenes. With a thickness of 2 μm, the PNF/Ti_3_C_2_T*_x_* hybrid showed an optimal performance with an areal capacity of 17.6 μA h cm^−2^, areal energy of 22.1 μW h cm^−2^, and an areal power of 1.5 mW cm^−2^. The contribution of the PNFs and MXenes in the hybrid electrode was distinctly studied, and the charge storage process was consisting of a faradaic part and a non-faradaic part. On account of the easily oxidized surface, the MXenes are always used as negative electrodes. However, in a recent study, the 3D porous Ti_3_C_2_T*_x_* MXene was reported to act as a positive electrode [99]. The surface of the MXene was covered by positively charged PANI to enhance the antioxidation of MXene, and polystyrene (PS) spheres were introduced as a sacrificial template to create a 3D open structure of the hybrid (Figure 7b). It was found that the hybrid exhibited an ultrahigh performance with specific capacitances of 1632 F cm^−3^ at 10 mV s^−1^, 1302 F cm^−3^ at 1000 mV s^−1^ and outstanding capacitance retention of 51% at a high scan rate of 5000 mV s^−1^, when the PANI content was 40%. The capacitance retention was 65.4% even the current density reached to 1000 A g^−1^. Based on the superior performance, an asymmetric device was fabricated to practically evaluate the feasibility of the PANI@M-Ti_3_C_2_T*_x_* electrode. The energy densities of the M-Ti_3_C_2_T*_x_*ǁPANI@M-Ti_3_C_2_T*_x_* were 50.6 mWh cm^−3^ at 1.7 W cm^−3^ and 24.4 mWh cm^−3^ at 127 W cm^−3^, respectively, and sharply increased to 1.381 kW cm^−3^ at 31 mWh cm^−3^ with a lessened cathode mass loading. Therefore, the open framework from 2D to 3D and the antioxidation of the MXenes was an innovative strategy to improve the capacitive properties.

The glycine molecules were successfully grafted onto the surface of the Ti_3_C_2_T*_x_* MXene via Ti–N bonding and showed enhanced capacitance [59]. The as-prepared d-Ti_3_C_2_T*_x_*/glycine reached 324 F g^−1^ at 10 mV s^−1^ and 140 F g^−1^ at 1 V s^−1^, respectively, and doubled the capacitance retention of the pristine Ti_3_C_2_T*_x_*. The LBL self-assembly approach could appropriately expand the interlayer spacing of the MXene sheets without weakening the interaction force between the MXenes and tris-(2-aminoethyl) amine [100]; hence, it was broadly employed. Wu et al. [101] reported a freestanding hybrid film through a combination of a decentralized conjugated polymer chain with Ti_3_C_2_T*_x_* MXene to enhance flexible cycle performance. The hybrid film not only exhibited an area capacitance of 284 mF cm^−2^ at 50 mA cm^−2^ and 100% capacitance retention after 10,000 cycles but also showed good stability during the 0–90° static bending test for 10,000 cycles. Another conjugated polymer with high charge mobility and tunable bandgap properties, (poly(9,9-dioctylfiuorene), PFO), has been proposed to be an electrochemically active material in 2017 [57]. Only the PFOs with charged nitrogen groups (denoted as P3) was attached to the negative Ti_3_C_2_T*_x_* layers due to the electrostatic interaction. The P3@Ti_3_C_2_T*_x_* showed an improved capacitance of 380 F g^−1^ at 2 mV s^−1^ and long-term stability. Later, the authors successfully functionalized the Ti_3_C_2_T*_x_* MXene by incorporating 1-aminoanthraquinone (AQ) [42]. The AQ-Ti_3_C_2_T*_x_* film showed a gravimetric capacitance of ~300 F g^−1^ at 5 mV s^−1^, which was higher than the pristine Ti_3_C_2_T*_x_* MXene. The outstanding capacitance could be attributed to enhanced charge transportation endowed by the AQ molecules intercalation and covalently grafting onto the surface of Ti_3_C_2_T*_x_* MXene surface. The performance of MXene/polymer hybrids for supercapacitors are summarized in Table 2.

### 4.2. MXene-Organic Hybrids for Flexible Metal-Ion Batteries

When getting into the field of metal-ion batteries, similar requirements as which in supercapacitors, including large specific surface area and excellent conductivity, are needed to be fulfilled. However, beyond these demands, the intrinsic electrochemical activity of the host materials is more crucial. Therefore, based on the abundant surface redox sites, the potential of MXene-organic hybrids in metal-ion batteries has been evaluated in recent years.

**Figure 8:**
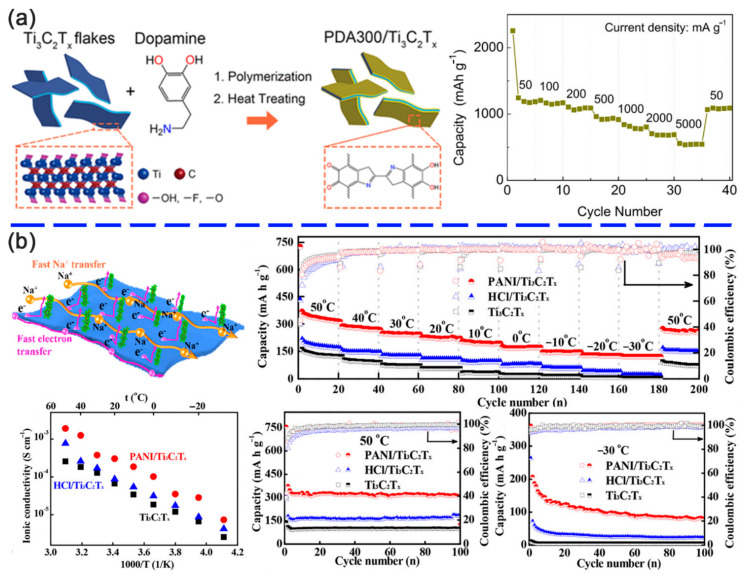
(**a**) Schematic diagram of the preparation route of PDA300/Ti_3_C_2_T*_x_* hybrid and the rate performance. Reproduced with permission [79]. Copyright 2018, American Chemical Society. (**b**) Sodium storage schematic diagram of PANI/Ti_3_C_2_T*_x_* and the electrochemical performance at different temperatures from −30 °C to +50 °C. Reproduced with permission [107]. Copyright 2020, American Chemical Society.

Siriwardane et al. [106] demonstrated that the pre-intercalated Ti_3_C_2_O_2_ by 1,4-benzoquinone (C_6_H_4_O_2_) or tetrafluoro-1,4-benzoquinone (C_6_F_4_O_2_) organic molecules facilitated the ion transportation within the interlayer space, thus resulting in a high lithium storage capacity and fast kinetics. Chen et al. prepared a Ti_3_C_2_T*_x_*/PEDOT hybrid by in situ polymerization triggered by electron transfer to improve lithium-ion storage capability [65]. On this basis, a vertically ordered mesoporous PDA was attached on the surface of Ti_3_C_2_T*_x_* MXene by Li and co-workers [81], which provided more channels for ion diffusion and electron transfer. A negligible volume expansion and the unvaried morphology of the mesoporous PDA and an ultrathin solid electrolyte interphase layer steadily surrounding the active material were found in the in situ transmission electron microscopy analysis. Dong et al. [79] reported PDA-derived material (denoted as PDA300) for the first time, which exhibited an ultrahigh capacity of 977 mAh g^−1^ at 50 mA g^−1^ compared with that of the original PDA (100 mAh g^−1^). A hybrid consisted of PDA300 and Ti_3_C_2_T*_x_* was fabricated (Figure 8a) and showed a high specific capacity of 1190 mAh g^−1^ at 50 mA g^−1^, excellent rate capability of 552 mAh g^−1^ at 5 A g^−1^, and good cycling stability (82% retained after 1000 cycles). Although the hybrid was not fabricated by in situ polymerization, the unsaturated carbon-carbon bonds in the PDA300 and the unique structure of the highly conductive Ti_3_C_2_T*_x_* MXene jointly enhanced the outstanding performance in lithium-ion batteries (LIBs). The same polymerization approach was also available to synthesize a 3D hierarchically porous PANI/Ti_3_C_2_T*_x_* network [107]. The mechanism of Na^+^ storage in the PANI/Ti_3_C_2_T*_x_* network is shown in Figure 8b. The larger interlayer space and negatively charged surface of Ti_3_C_2_T*_x_* nanosheets generated plenty of channels for Na^+^ diffusion. Meanwhile, the 2D electron-transfer platforms provided by highly conductive Ti_3_C_2_T*_x_* nanosheets guaranteed the excellent electrical conductivity of the PANI/Ti_3_C_2_T*_x_* network. Interestingly, the combination of MXene flakes and PANI generated a tough but pliable network to accommodate the volume change during Na^+^ diffusion, which also had good tolerance to temperature change. Therefore, the PANI/Ti_3_C_2_T*_x_* network exhibited an excellent capacity at the broad temperature range from −30 °C to +50 °C. Furthermore, the PANI/Ti_3_C_2_T*_x_* network also presented an excellent specific capacity of 254 mA h g^−1^ after 100 cycles at 100 mA g^−1^ and an ultrastable cycling capacity of 135.4 mA h g^−1^ at a high current density of 2000 mA g^−1^ after 10,000 cycles. The full cell assembled by PANI/Ti_3_C_2_T*_x_* anode and Na_3_V_2_(PO4)_3_ cathode delivered a high discharge capacity of 140.1 mA h g^−1^ after 100 cycles.

### 4.3. MXene-Organic Hybrids for Flexible Sensor

**Figure 9:**
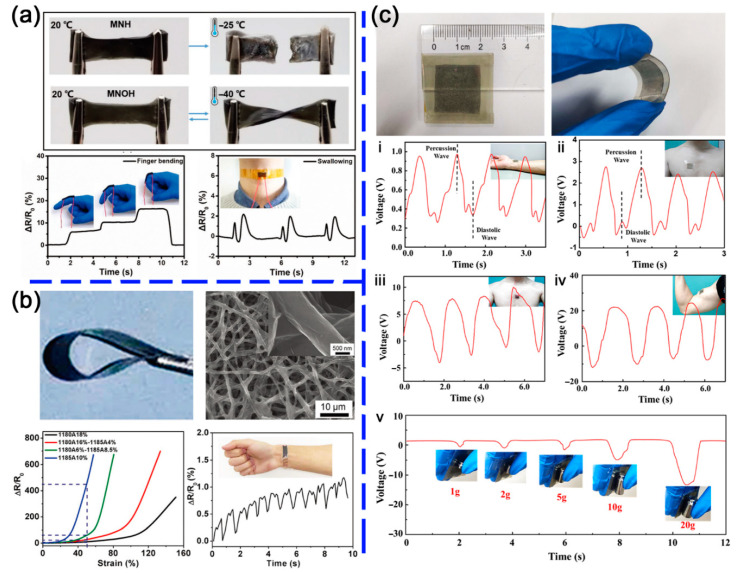
(**a**) Top: photographs of the low-temperature tolerant behavior of MNH and MNOH; Bottom: relative resistance variation of MNOH-based strain sensors after being stored at −40 °C for 6 h in response to finger bending with different angles and saliva swallowing. Reproduced with permission [87]. Copyright 2019, WILEY-VCH. (**b**) Top: photographs of flexible network-M/P mat and SEM images of the network-M/P mat with wrinkled surfaces. Bottom: relative resistance variation-strain curves of network-M/P mat sensors with different elastomer components and monitoring of pulse beat. Reproduced with permission [108]. Copyright 2019, Royal Society of Chemistry. (**c**) Top: images and flexibility demonstration of the triboelectric tactile sensor. Bottom: real-time V-t curves of the tactile sensors for monitoring (i) pulse, (ii) heartbeat, (iii) breath, (iv) flexion and extension of biceps, and (v) grab of five different weights. Reproduced with permission [109]. Copyright 2020, Elsevier B.V.

When turning to sensors, benefiting from the anisotropic conductivity of MXenes, the electroresponse of MXene-based hybrids can be easily regulated by changing the testing direction. However, also due to the in-plane metallic conductivity of MXene sheets, the deformation and variation of surface adsorption species show a limited impact on the internal resistance. Therefore, an interfacial engineering strategy towards tuning the electrical properties of the entire material system becomes more powerful. In addition, although the coating of MXenes on various 3D skeletons can generate a large variety of MXene-based hybrid sensors, here we only focus on the MXene-organic hybrids that are consisting of certain organic components and through strong interfacial bonding. Liao and co-works [87] fabricated a flexible wearable strain sensor assembled with Ti_3_C_2_T*_x_* MXene nanocomposite organohydrogel (MNOH), which was prepared by immersing MXene nanocomposite hydrogel (MNH) in ethylene glycol solution (Figure 9a). The MNOH maintained flexibility and reversible bending capability and successfully lighted a LED at an extremely low temperature of −40 °C on account of the strong hydrogen bonds and excellent conductivity, while the fracture occurred, and the LED were not worked when the MXene hybrid hydrogel was at −25 °C. Meanwhile, the MNOH also owned a good self-healing capacity and showed no fracture even at a stretching condition, and the lighted LED further demonstrated the self-healing performance. In addition, the MNOH-based strain sensor could monitor human activities by connecting to a wireless transmitter, such as finger bending with different angles and saliva swallowing. Besides the flexible, self-healing, and wearable properties, long-time storage, and superior self-adhesive Ti_3_C_2_T*_x_* MNOH were manufactured by Wu’s team [88]. After been stored for 10 days at 20 °C and 55% humidity, the organohydrogel retained 90% moisture and showed no shape change and no fracture upon twisting or bending. The MNOH supported a weight of 105 g without any adhesives and was used repeatedly with no significant loss of the adhesion strength. The muscle movement caused by breathing was monitored via the sensor attached to the chest, which further validated its high sensitivity. High-strength also could be associated with hybrid double-network hydrogens [85], which could be stretched to over 14 times of the pristine length and achieve a 0.4 MPa tensile strength meanwhile reserve the self-healing ability, temperature-sensitive ability, and excellent conductivity. A network-structured Ti_3_C_2_T*_x_* MXene/PU mat (network-M/P mat) not only provided a high sensitivity with the gauge factor up to 228 and a lower detection limit with 0.1% but also exhibited a large and tunable sensing range up to 150% [108] (Figure 9b), excellent stability over 3200 cycles and multiple functions towards strain from various direction and deformation. Cai et al. recently developed a PDMS/Ti_3_C_2_T*_x_* MXene hybrid as electronic skin for intelligence daily health monitoring [109]. As a common flexible substrate, the PDMS can be easily patterned with surface wrinkles. When blended with MXenes, the hybrid presented a sensitive electroresponse to the applied pressure and can be assembled as a self-powered tactile sensor. As shown in Figure 9c, for monitoring (i) pulse, (ii) heartbeat, (iii) breath, (iv) flexion and extension of biceps, and (v) grab of five different weights, the sensor revealed different waveforms for different cases, which provided more information for medical professionals to monitor patient status in real time.

**Figure 10:**
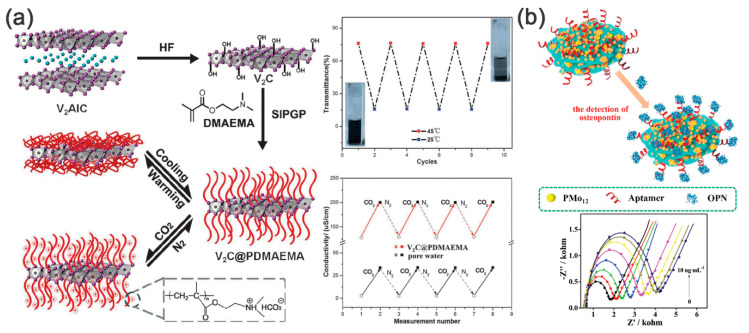
(**a**) Left: schematic representation of preparing V_2_C@PDMEMA smart hybrid systems. (C—off-white, V—purple, Al—blue). Right-top: transmittance change in the V_2_C@PDMEMA suspension during heating-cooling cycles between 25 °C and 45 °C. Inset: digital images for the dispersion status of V_2_C@PDMEMA below and above the LCST. Right-bottom: cyclic changes in the conductivity of pure water and V_2_C@PDMEMA aqueous dispersion measured at 25 °C during CO_2_ uptake and subsequent CO_2_ release. Reproduced with permission [49]. Copyright 2015, Royal Society of Chemistry. (**b**) Top: schematic diagram of the aptasensor fabrication based on PPy@Ti_3_C_2_T*_x_*/PMO_12_ for OPN detection. Bottom: EIS responses of the OPN/Apt/PPy@Ti_3_C_2_T*_x_*/PMo_12_/AE for the detection of OPN with different concentrations (0.05, 0.5, 5, 50, 500, 5000, 10,000 pg mL^−1^). Reproduced with permission [110]. Copyright 2020, Elsevier B.V.

Chen [49] reported that a V_2_C@(poly(2-(dimethylamino)ethyl methacrylate), PDMEMA) hybrid could undergo hydrophobic-hydrophilic transitions in water for the first time, and the transmittance and conductivity of the hybrid were changed along with external temperature due to the thermal behavior of PDMEMA (Figure 10a). The opposite state of the PDMEMA chains at different temperatures resulted in the change of the V_2_C@PDMEMA suspension in water, with the corresponding transmittance changed from 15% to 75%. The change of conductivity of 76 μS cm^−1^ was also monitored in the V_2_C@PDMEMA suspension by cycled bubbling CO_2_ and N_2_ as CO_2_ stimulation induced the uncharged-charged transitions of PDMEMA (Figure 10a). Later, Tran et al. [32] found that the existence of the covalent bonding between the MXene and PDMEMA and investigated the mechanism by density functional theory. The hybrid also presented a switchable behavior at below or above the lower critical solution temperature of PDMEMA. The DFT further elucidated that the polymer state (swelling or shrinking) at different temperatures changed the interlayer distance of the MXene, which resulted in the varied conductivity.

The sensors not only respond to temperature or physical parameters such as strain but also can be triggered by some chemical molecules. For example, An et al. [111] firstly found that Ti_3_C_2_T*_x_* MXene/polyelectrolyte multilayer films prepared by LBL assembly could immediately respond to H_2_O molecules. The intercalation or deintercalation of H_2_O changed the interlayer space between two MXene layers and resulted in a changed tunneling resistance between MXene sheets. Later, Neampet [112] and co-workers further enriched the sensor applying range by preparing a lactate oxidase immobilized on Pt//PANI/Ti_3_C_2_T*_x_* MXene hybrid for a modified screen-printed carbon electrode, which shows good response to H_2_O_2_ and lactate. Besides, oxygen-containing volatile organic compounds, such as ethanol and acetone, could be detected by chemosensitive sensors, which were made by fluoroalkyl silane molecules modified Ti_3_C_2_T*_x_* [113]. The synergy of the strong adsorption energy of ethanol on Ti_3_C_2_T*_x_*–F and the local structure deformation contributed to the high sensitivity, good repeatability as well as long-term stability. By utilizing the conductivity of MXenes, Li et al. presented a flexible PANI/Ti_3_C_2_T*_x_* MXene hybrid for selective NH_3_ gas monitoring [114]. The in situ polymerization of PANI with Ti_3_C_2_T*_x_* MXene generated a homogeneous Schottky junction. At this interface, Ti_3_C_2_T*_x_* induced an improved protonation of PANI, which resulted in high selectivity. As reported by Zhou and the co-workers, the PPy@Ti_3_C_2_T*_x_* hybrid can be a good platform for osteopontin detection (Figure 10b) [110]. After loading the phosphomolybdic acid (PMo_12_), the final hybrid delivered an extremely low detection limit of 0.98 fg mL^−1^, as well as high selectivity and stability. The superior performance was ascribed to the abundant surface active sites (Ti^4+^ from MXenes and Mo^4+^ from PMo_12_) and the conducting network of PPy@Ti_3_C_2_T*_x_* hybrid. When directly blended with polymer, the MXene-based hybrids also can be a good candidate for advanced sensors. Recently, Wang et al. reported a PEDOT:PSS/Ti_3_C_2_T*_x_* hybrid for room-temperature methanol detector [68]. As a p-type semiconductor, the adsorption of electron-donating gas neutralized the holes in PEDOT:PSS, thus raising the resistance. While for the PEDOT:PSS/Ti_3_C_2_T*_x_* hybrid, the conductive mechanism changed to a summation of a mixed pathway, in which the MXene-MXene connection was the easiest to destroy. Therefore, the PEDOT:PSS/Ti_3_C_2_T*_x_* hybrid with a suitable polymer/MXene ratio revealed a large response to methanol.

### 4.4. MXene-Organic Hybrids for Other Applications

The diversified properties of the modified MXenes have also been applied in the fields of photocatalysis [115], molecular separation [78,116], seawater desalination [29], flame retardant [48], and others. Li et al. [115] prepared a modified Ti_3_C_2_T*_x_* MXene by a chlorophyll derivative called zinc methyl 3-devinyl-3-hydroxymethyl-pyropheophorbide (Chl), and the hybrid exhibited a high HER performance with 52 ± 5 μmol h^−1^ g cat^−1^ at the optimal Chl/Ti_3_C_2_T*_x_* ratio. The aggregated exciton transfer in Chl and the resultant charge separation at the interface of Chl/Ti_3_C_2_T*_x_* after the efficient light-harvesting improved performance together. Liu et al. [117] found that a 100 nm thickness of Ti_2_CT*_x_*-(hyperbranched poly-ethyl enimine, HPEI)/(tri-mesoyl chloride, TMC) membrane with ordered 2D nanochannels could extract 99 wt% water from the 10 wt% water/isopropanol mixtures. Shen et al. [116] prepared a thinner nanofilm about 10 nm to separate gas molecules. The chemically tuned Ti_3_C_2_T*_x_* MXene allowed CO_2_ permeation, while the H_2_ selective transport in the pristine MXene nanofilms. Zhao et al. [29] proposed a hydrophobic MXene membrane consisting of trimethoxy (1H,1H,2H,2H-perfluorodecyl) silane (PFDTMS) modified Ti_3_C_2_T*_x_* nanosheets and commercial filter membrane to issue salinity water desalination. The fabricated membrane not only possessed high-efficiency with a solar evaporation rate of 1.31 kg m^−2^ h^−1^ and 71% solar steam conversion efficiency, but also stably worked over 200 h. A poly(diallyldimethylammoniumchloride) (PDDA) modified Ti_3_C_2_T*_x_* MXene adding to thermoplastic polyurethane matrix obviously decreased the peak heat-release rate of 50% and lessened total smoke production of 47% [48].

## 5. Outlook

In consideration of the fascinating electric, mechanical properties, and processability of MXene-organic hybrids, the combination of MXene with suitable organic counterparts will offer a broad space for fabricating flexible electronics. To get more specific guidance for materials design, a deeper understanding of the interaction between MXene and the organic components is necessary. In this review, we mainly focus on the surface characters of pristine MXene and the accompanying physicochemical interaction between MXene and the organic components in the hybrid. The applications of MXene-organic hybrids in the fields of flexible devices are also briefly summarized.

There is no doubt that the research on MXene-organic hybrids is still at its early stage, but the rapid development of wearable devices for intelligent sensing, health monitoring, and portable display will boost the demands of relevant materials in the near future. Moreover, although the importance of interfacial behaviors between the MXene and the organic component has been widely concerned, there is still plenty of work that needs to be done, especially concerning the detailed roles of different surface terminal groups on the interaction with organic molecules and the mechanism of carriers’ transition on the MXene-organic interface. We believe that with the highly compatible interface, the MXene-organic hybrids will fully integrate the advantages from both MXene and organic molecules, thus opening a new territory for next-generation flexible wearing devices.

## Figures and Tables

**Table 1:** Summary of the interactions between MXene and organic species.

Interaction Type	Typical Modifier	Binding Forms
Covalent interaction	Silane	^a^ Si–O–M
	Diazonium salts	C–O–M
	Alkyl phosphoric acid	P–O–M
Electrostatic interaction	Alkyl ammonium salts	^b^ R_4_N⊕ ⊝T–M
	Amino acids	–H_3_N⊕ ⊝T–M
	PFO	R_4_N⊕ ⊝T–M
	PANI	–H_3_N⊕ ⊝T–M
	PEDOT:PSS	SO_3_H⊕ ⊝T–M
Hydrogen bonds	Pyrrole/PPy	NH···O–M
	Dopamine/PDA	OH···O–M
	PAM	CONH_2_···O–M
	PVA	OH···O–M
	PSS	SO_3_H···O–M

^a^ M represents a metal atom from the surface layer of MXenes. ^b^ R represents any alkyl group.

**Table 2:** Comparison of MXene/polymer hybrids for supercapacitors.

MXene Type	Capacitance@Rate	Electrolyte	Cycle Number	Cycling Stability (%)	Refs.
PDT/Ti_3_C_2_T*_x_*	284 mF cm^−2^@50 mA cm^−2^	0.5 M H_2_SO_4_	10,000	100	[101]
PPy/l-Ti_3_C_2_T*_x_*	203 mF cm^−2^	0.5 M H_2_SO_4_	20,000	100	[77]
PPy/Ti_3_C_2_T*_x_* (1:2)	1000 F cm^−3^@5 mV s^−1^	1 M H_2_SO_4_	25,000	92	[76]
Mo_1.33_C/PEDOT:PSS	1310 F cm^−3^@2 mV s^−1^	1 M H_2_SO_4_	10,000	90	[71]
Ti_3_C_2_T*_x_*/P-100-H	1065 F cm^−3^@2 mV s^−1^	1 M H_2_SO_4_	10,000	96	[70]
GMP	635 F g^−1^@1 A g^−1^	1 M H_2_SO_4_	10,000	97.54	[102]
Ti_3_C_2_T*_x_*/PDA	715 mF cm^−2^@2 mV s^−1^	1 M H_2_SO_4_	10,000	95.5	[103]
P3@Ti_3_C_2_T*_x_*	380 F g^−1^@ 2 mV s^−1^	1 M H_2_SO_4_	10,000	98	[57]
MXene/PANI	556.2 F g^−1^@0.5 A g^−1^	1 M H_2_SO_4_	5000	91.6	[64]
MXene/PANI	503 F g^−1^@2 mV s^−1^	3 M H_2_SO_4_	10,000	98.3	[104]
d-Ti_3_C_2_T*_x_*/glycine	324 F g^−1^@ 10 mV s^−1^ 140 F g^−1^@ 1000 mV s^−1^	3 M H_2_SO_4_	20,000	~100	[59]
PANI@M-Ti_3_C_2_T*_x_*	1632 F cm^−3^@10 mV s^−1^	3 M H_2_SO_4_	20,000	85.7	[99]
Ti_3_C_2_T*_x_*@PPy NW	610 F g^−1^@0.5 A g^−1^	3 M KOH	20,000	100	[89]
MXene/PANI (1:3)	592 F g^−1^@ 0.5 A g^−1^	7 M KOH	10,000	95.3	[105]

## Data Availability

The data presented in this study are available in cited references.
